# Understanding patients' self-management after enterostomy: knowledge, attitudes, and practices in a cross-sectional study

**DOI:** 10.3389/fpubh.2026.1681498

**Published:** 2026-03-18

**Authors:** Hua Yang, Runhong Ni, Nan Qiao, Zhijing Wang

**Affiliations:** 1 Department of Gastrointestinal Surgery, China-Japan Friendship Hospital, Beijing, China; 2 Department of Colorectal Surgery, China-Japan Friendship Hospital, Beijing, China

**Keywords:** attitude, enterostomy, knowledge, patient, practice, self-management

## Abstract

**Background:**

Few studies have explored the knowledge, attitudes, and practices (KAP) related to self-management among post-enterostomy patients, particularly in China. Such understanding is crucial for developing targeted interventions that improve long-term recovery, quality of life, and healthcare resource utilization. This study aimed to fill this gap by examining KAP levels and their interrelationships in this population.

**Methods:**

A cross-sectional survey was conducted at the China-Japan Friendship Hospital between March 15 and May 15, 2024, using a structured questionnaire with confirmed reliability (Cronbach's α = 0.825, 0.748, and 0.770, respectively) and validity.

**Results:**

A total of 497 valid responses were analyzed. The median scores for KAP were 12.00 (IQR: 11.00–13.00), 41.00 (IQR: 35.00–47.00), and 70.00 (IQR: 63.00–77.00), respectively. Path analysis revealed that knowledge improved attitudes, which subsequently promoted better self-management practices. Bootstrapping analysis confirmed a significant indirect effect of knowledge on practice through the mediating role of attitudes (β = 0.262, 95% CI [0.089, 0.451]).

**Conclusion:**

This study provides new evidence that attitudes mediate the relationship between knowledge and self-management practices among post-enterostomy patients. It highlights the scientific value of applying the KAP framework to this population and underscores the need for attitude-focused education, structured follow-up, and psychosocial support. Future research should explore longitudinal interventions to sustain long-term self-managemente improvements.

## Introduction

An enterostomy is surgically created by bringing the bowel to the abdominal surface, a procedure known as enterostomy, which connects the intestinal lumen with the abdominal wall. This surgical intervention is primarily used for conditions such as colorectal cancer, inflammatory bowel disease, and trauma. As a crucial treatment for colorectal cancer, enterostomy effectively addresses postoperative defecation issues and contributes to improved survival rates (1). In the United States, it is estimated that between 650,000 and 730,000 individuals live with a permanent enterostomy (2).

In China, nearly 100,000 patients with colorectal cancer opt for enterostomy surgery annually (3). Although lifesaving for most, enterostomy surgery significantly impacts patients' normal bowel functions, thereby affecting their everyday life and social interactions (4–7). Similar challenges have been observed globally; for instance, a longitudinal study in the United Kingdom found that enterostomy-forming surgery alters individuals' social world, reducing social confidence and autonomy (8). Likewise, a cross-national survey involving more than 2,200 rectal-cancer survivors across five European countries revealed that enterostomy-related problems such as leakage, odor, and skin irritation remain common and restrict daily activities (9). Complementary patient-survey data from the UK further emphasize the ongoing need for structured rehabilitation and education programmes after enterostomy formation (10). These findings underscore the global relevance of investigating knowledge, attitudes and practices toward post-enterostomy self-management.

The psychosocial health of patients with enterostomies is notably affected during the hospitalization and post-discharge transition periods. Evidence suggests that patients experience greater social isolation post-discharge than during hospitalization, which manifests as poor body image, low self-esteem, severe negative emotions, and diminished psychosocial adaptation (2, 11). These issues are compounded by postoperative complications, coping self-efficacy, the level of social support, family living situation, and other factors, contributing significantly to the challenges patients face in adapting to life with an enterostomy and to their overall quality of life (12–14). Beyond psychosocial challenges, healthcare system support and economic factors also critically influence post-enterostomy self-management. In many healthcare settings, disparities in access to enterostomy care supplies, continuity of professional follow-up, and insurance reimbursement policies contribute to unequal self-care capabilities and adjustment outcomes (2, 15). Moreover, the financial burden associated with enterostomy maintenance, including costs of appliances, accessories, and regular consultations, has been identified as a significant barrier to sustained self-management, especially among patients with limited health coverage. Addressing these systemic and economic dimensions is essential for understanding the broader determinants of self-management behavior.

The Knowledge-Attitude-Practice (KAP) model posits that knowledge forms the foundation for behavior change, with attitudes and beliefs acting as the driving forces behind this change (16). Practice modification occurs in three stages: acquiring knowledge, forming attitudes/beliefs, and developing practices (17). However, knowledge alone does not guarantee behavior change; it must first influence perceptions, which then drive changes in practice (18). This study addresses the gap in understanding the specific determinants of self-management practice among post-enterostomy patients in China. While self-management's importance is well-documented, the mechanisms through which knowledge influences practice, particularly the role of attitudes in this process, remain poorly understood. Recent reviews highlight significant evidence gaps in optimal ostomy care, particularly regarding patient-centered outcomes and behavioral factors (19). This lack of mechanistic understanding hinders the development of targeted and effective educational interventions. This lack of understanding impedes the development of effective educational interventions. This study aims to provide an evidence-based framework for designing more effective support programs that go beyond knowledge dissemination, fostering positive attitudes and improving self-care practices and long-term outcomes.

## Material and methods

### Study design and subjects

This cross-sectional survey was conducted at the China-Japan Friendship Hospital from March 15, 2024, to May 15, 2024, focusing on patients who had undergone enterostomy within the past decade. Eligible participants were identified through the hospital's outpatient database and selected using a simple random sampling procedure, following procedures described in previous cross-sectional studies on surgical self-management (20, 21). Specifically, patients attending the enterostomy outpatient or gastrointestinal surgery clinics during the study period were invited to participate. The diagnosis of enterostomy was verified by the attending colorectal or gastrointestinal surgeon based on the hospital's standardized clinical protocol. The study received ethical approval from the Clinical Research Ethics Committee of the China-Japan Friendship Hospital, and all participants provided written informed consent. To ensure confidentiality, all questionnaires were completed anonymously and coded numerically, with data stored securely on password-protected hospital servers accessible only to the research team. A total of 504 patients met the eligibility criteria, of whom 497 completed valid questionnaires and were included in the final analysis, yielding a validity rate of 98.61%.

Inclusion criteria included: (1) patients with an enterostomy; (2) duration of enterostomy greater than three months. The threshold of enterostomy duration greater than three months was chosen because preliminary clinical practice indicates that basic enterostomy self-care skills and adaptation tend to stabilize after the first three months post-surgery (3, 11) consent to participate in the survey. Exclusion criteria included: (1) individuals completely unable to care for themselves. Patients who were completely unable to care for themselves were excluded because the focus of this study is on self-management behaviors; inclusion of such individuals could bias results toward lower practice outcomes and not reflect the target population of able self-managers; (2) individuals with impaired consciousness impairing normal communication.

### Ethical clearance

The study protocol was reviewed and approved by the Clinical Research Ethics Committee of the China-Japan Friendship Hospital (Approval No. 2024-KY-082). A research permit was obtained prior to data collection. All participants were fully informed about the study objectives, procedures, and their right to withdraw at any time without penalty. Written informed consent was obtained from all participants before enrollment. The study was conducted in accordance with the ethical principles outlined in the Declaration of Helsinki (revised 2013) and complied with institutional and national research ethics standards.

### Questionnaire introduction

The questionnaire was developed referencing previously published literature (20, 21). The initial questionnaire was piloted among 43 respondents. Internal consistency was evaluated by calculating Cronbach's α coefficients for each subscale separately. The coefficients were 0.825 for the knowledge dimension, 0.748 for the attitude dimension, and 0.770 for the practice dimension, indicating acceptable reliability for each construct. Content validity was ensured by adapting items from relevant literature to the clinical and cultural context of the study population. As this study serves as a preliminary, descriptive investigation, no formal exploratory factor analysis (EFA) or construct validation was performed to establish a latent factor structure.

The questionnaire included four sections: demographics, knowledge (13 items, scored 0–13), attitude (10 items, Likert scale 1–5, total 10–50), and practice (16 items, Likert scale 1–5, total 16–80). Scores equal to or above 70% were considered adequate (22).

### Questionnaire distribution and quality control

The study targeted patients attending enterostomy outpatient or gastrointestinal surgery of our hospital, including members of the hospital's enterostomy group and enterostomy patients under the care of the researchers. Questionnaires were primarily distributed within the hospital's gastrointestinal and surgical departments. Surveys were conducted mainly in these clinics, with gastroenterologists and surgical nurses assisting in the process. Prior to the survey, training sessions were held to enhance knowledge dissemination related to gastrointestinal surgery and improve communication with patients.

### Statistical analysis

The statistical analysis software used was SPSS 27 & AMOS 26 (IBM, Armonk, New York, USA). Continuous variables not conforming to a normal distribution were presented as medians and interquartile ranges (IQR), while categorical data were expressed as frequency (percentage). To compare differences in knowledge (K), attitude (A), and practice (P) scores across different demographic characteristics, the Mann-Whitney *U*-test and the Kruskal-Wallis *H*-test were utilized for group comparisons. Spearman's rank correlation coefficient was employed to analyze the relationships between knowledge, attitude, and practice scores. Multivariate linear regression was conducted to identify factors influencing practice scores. Path analysis was employed to assess the hypotheses: (1) knowledge directly influences attitude and practice; and (2) attitude directly affects practice. To robustly test the mediation hypothesis, we used bootstrapping with 5,000 resamples to obtain bias-corrected 95% confidence intervals (CIs) for the indirect effects. The path model was saturated (degrees of freedom = 0); therefore, global fit indices (e.g., CFI, TLI, RMSEA) are not applicable to the saturated model and are not reported. A two-sided *P* < 0.05 was considered statistically significant.

## Results

Initially, the study collected 504 samples. After excluding seven cases with anomalous responses related to occupation, 497 valid questionnaires remained, yielding a validity rate of 98.61%. Of these, 307 (61.77%) were male, with a mean age of 65.17 ± 8.73 years. A total of 232 respondents (46.68%) resided in urban areas, 180 (36.22%) had attained a junior high school education, and 225 (45.27%) were retired. Most respondents (400, 80.48%) procured enterostomy supplies through medical insurance. Additionally, 234 respondents (47.08%) were fully capable of managing their own enterostomy care, and 246 (49.50%) had had their enterostomy for more than 24 months. Among the respondents, 214 (43.06%) had a temporary enterostomy, with 37 (7.44%) undergoing restoration 6–12 months post-surgery, and 173 (34.81%) had plans for future enterostomy restoration. The median scores for KAP were 12.00 (IQR: 11.00–13.00), 41.00 (IQR: 35.00–47.00), and 70.00 (IQR: 63.00–77.00), respectively. Place of residence, education, occupation, income, and medical insurance type all showed significant effects on patients' KAP (*P* < 0.05). Rural residents and farmers demonstrated higher knowledge and more positive attitudes toward self-management. Individuals with elementary education or below were more active in self-care practices. Those covered only by social medical insurance achieved higher KAP scores than patients with additional commercial insurance. Longer enterostomy duration (over 24 months) was also associated with better KAP (Table 1).

**Table 1:** Demographic characteristics and KAP scores.

**Variables**	**[*N* (%)]**	**Knowledge, median (IQR)**	***P***	**Attitude, median (IQR)**	***P***	**Practice, median (IQR)**	***P***
**Total score**	*N* = 497	13 (1)	–	45 (7)	–	75 (7)	–
**Age**	71 (11)	–	–	–	–	–	–
**Gender**							
Male	307 (61.77)	12 (1)	0.244	43 (15)	0.564	73 (11)	0.731
Female	190 (38.23)	12 (1.25)	–	43 (14)	–	74 (12.25)	–
**Marital status**							
Married	453 (91.15)	12 (1)	0.072	43 (12)	0.759	73 (11)	0.954
Single or divorced or widowed	44 (8.85)	13 (1)		45 (16.5)		75 (13)	
**Residence**							
Rural	102 (20.52)	12 (0)	0.044	45 (11)	<0.001	75 (20.25)	0.908
Urban	232 (46.68)	13 (2)		45 (12)		75 (8)	
Suburbs	163 (32.80)	12 (0)		43 (19)		69 (18)	
**Education**							
Elementary school and below	81 (16.30)	12 (1)	0.011	45 (10)	<0.001	75 (16)	<0.001
Junior high school	180 (36.22)	12 (1)		43 (11)		64 (20)	
High school/technical secondary school	69 (13.88)	13 (2)		48 (15)		75 (2)	
College/undergraduate	150 (30.18)	13 (2)		45 (17)		75 (6)	
Master's degree and above	17 (3.42)	11 (1)		28 (10)		67 (3)	
**Occupation**							
Farmer	93 (18.71)	12 (1)	<0.001	45 (10)	<0.001	75 (18)	<0.001
Worker	64 (12.88)	13 (2)		48 (18)		75 (8)	
White collar	75 (15.09)	11 (1)		33 (11)		69 (6)	
Retire	225 (45.27)	12 (1)		45 (5)		75 (13)	
Other	40 (8.05)	13 (1)		45 (14)		75 (6)	
**Medical insurance type**							
Only social medical insurance	480 (96.58)	12 (1)	<0.001	43 (15)	<0.001	75 (11)	0.006
Both social medical insurance and commercial medical insurance	17 (3.42)	11 (2)		28 (18)		69 (8.5)	
**Household monthly per capita income**							
<2000	101 (20.32)	12 (0)	<0.001	45 (11)	<0.001	75 (21.5)	0.002
2000–4999	57 (11.47)	13 (0)		48 (9.5)		75 (15)	
5000–9999	267 (53.72)	12 (1)		45 (8)		75 (11)	
≥10000	72 (14.49)	11 (1)		28 (0)		69 (1)	
**The cost of purchasing enterostomy supplies mainly comes from**							
Medical insurance	400 (80.48)	12 (1)	0.171	43 (12)	<0.001	73 (11)	0.101
Own expense	97 (19.52)	12 (0)		45 (10.5)		75 (16)	
**Can I take care of my enterostomy now?**							
Completely unable to care	40 (8.05)	13 (2)	<0.001	48 (12)	0.272	75 (17)	0.007
Partially care	223 (44.87)	13 (1)		45 (7)		75 (8)	
Completely care	234 (47.08)	12 (0)		40 (15)		73 (13)	
<6 months	66 (13.28)	11 (0)	<0.001	28 (7.25)	<0.001	69 (2)	<0.001
6–12 months	79 (15.90)	12 (1)		39 (17)		69 (15)	
13–24 months	106 (21.33)	12 (1)		43 (11)		64 (20)	
>24 months	246 (49.50)	13 (1)		45 (8)		75 (2)	
**Enterostomy type**							
Temporary enterostomy	214 (43.06)	12 (2)	0.052	40 (12)	<0.001	70 (11)	<0.001
Permanent enterostomy	283 (56.94)	12 (1)		45 (13)		75 (13)	
**A survey of patients with temporary enterostomy (protective enterostomy)**							
**Has the enterostomy been restored?**							
Yes	37 (7.44)	13 (1)	–	45 (5)	–	75 (2)	–
No	177 (35.61)	12 (2)	–	40 (14)	–	67 (11)	–
**How long after surgery was the restoration performed?**							
Within 3 months after surgery	0	–	–	–	–	–	–
3–6 months after surgery	0	–	–	–	–	–	–
6–12 months after surgery	37 (7.44)	13 (1)	–	45 (5)	–	75 (2)	–
1 year after surgery	0	–	–	–	–	–	–
**Is there any restoration plan?**							
Yes	173 (34.81)	12 (2)	–	40 (14)	–	69 (11)	–
No	4 (0.80)	10 (2)	–	26.5 (19)	–	51 (24)	–

The knowledge dimension revealed that the highest correctness rates were for the items: “If the enterostomy is cut too low, it can easily get stuck in the intestine, causing bleeding or ischemic necrosis” (K10) at 99.80%, and “If the enterostomy chassis is cut too large, leakage may occur, causing fecal aqueous dermatitis” (K9) at 99.60%. The lowest correctness rates were observed for “enterostomy patients cannot fly because they are under too much pressure and cannot clear their bowels in time” (K7) at 48.49%, and “Do you know about the common complications of enterostomy and surrounding areas?” (K12) at 80.48% (Supplementary Table 1).

Attitudinal responses indicated that 67.61% strongly disagreed with the concern that others might fear contracting their disease through contact (A3), and 66.4% strongly disagreed that their disease led to decreased respect from others (A2). Regarding concerns about unauthorized disclosure of their condition (A5), 31.79% were neutral. Notably, 19.11% felt isolated from healthy individuals (A1), and 14.69% felt perceived uselessness and reported that changes in appearance had impacted their social relationships (Supplementary Table 2).

Responses in the practice dimension showed a positive trend, with 77.06% willing to share their experiences (P7), 74.65% knowledgeable about cleaning the enterostomy bag (P5), and 67.2% consistently managing their emotions related to enterostomy care (P15). However, 10.06% never and 8.25% seldom changed the enterostomy bag and accessories independently (P2), highlighting a need for further attention in this area (Supplementary Table 3).

Correlation analysis identified significant positive correlations between knowledge and attitudes (*r* = 0.520, *P* < 0.001), knowledge and practices (*r* = 0.324, *P* < 0.001), and attitudes and practices (*r* = 0.784, *P* < 0.004) (Table 2).

**Table 2:** Correlation analysis.

**Dimension**	**Knowledge**	**Attitude**	**Practice**
Knowledge	Knowledge	**–**	**–**
Attitude	0.520 (*P* < 0.001)	1	**–**
Practice	0.324 (*P* < 0.001)	0.784 (*P* = 0.004)	1

Multivariate linear regression indicated that knowledge score (Beta = 1.221, 95% CI: [0.566–1.877], *P* < 0.001), attitude score (Beta = 0.726, 95% CI: [0.637–0.815], *P* < 0.001), junior high school education (Beta = −12.621, 95% CI: [-16.876 −8.365], *P* < 0.001), Master's degree or higher (Beta = 7.461, 95% CI: [1.473–13.449], *P* = 0.015), employment status as a worker (Beta = −5.747, 95% CI: [-8.836 −2.658], *P* < 0.001), and ability to fully care for enterostomy (Beta = 6.493, 95% CI: [4.036–8.950], *P* < 0.001) were independently associated with practice (Table 3).

**Table 3:** Univariate and multivariate analysis of practice.

**Variable**	Univariate linear regression		Multivariate linear regression	
	**Beta (95%CI)**	***P***	**Beta (95%CI)**	***P***
Knowledge score	3.616 (2.910–4.322)	<0.001	1.221 (0.566–1.877)	**<0.001**
Attitude score	0.630 (0.555–0.706)	<0.001	0.726 (0.637–0.815)	**<0.001**
Age	−0.032 (−0.124–0.061)	0.499	–	–
**Gender**				
Male	0.626 (−1.029–2.282)	0.458	–	
Female	Ref			
**Marital status**				
Married	Ref	0.244	–	
Single or divorced or widowed	−1.682 (−4.512 – 1.148)		
**Residence**				
Rural	ref	–	–	
Urban	1.129 (−1.003 – 3.261)	0.299		
Suburbs	0.714 (−1.552 – 2.980)	0.536		
**Education**				
Elementary school and below	Ref	–	ref	–
Junior high school	−5.049 (−7.395–2.704)	<0.001	−12.621 (−16.876–8.365)	**<0.001**
High school/technical secondary school	−1.078 (−3.950–1.793)	0.461	−2.942 (−7.865–1.980)	0.241
College/undergraduate	−1.069 (−3.486–1.348)	0.385	−0.383 (−5.253–4.488)	0.877
Master's degree and above	−3.638 (−8.314–1.039)	0.127	7.461 (1.473–13.449)	**0.015**
**Occupation**				
Farmer	−1.580 (−4.957–1.796)	0.358	−1.787 (−5.803–2.230)	0.383
Worker	−2.841 (−6.440–0.759)	0.122	−5.747 (−8.836–2.658)	**<0.001**
White collar	−4.568 (−8.065–1.072)	0.011	−1.903 (−4.688–0.883)	0.180
Retire	−2.431 (−5.495–0.634)	0.120	−1.195 (−3.252–0.863)	0.254
Other	Ref	–	ref	–
**Medical insurance type**				
Only social medical insurance	Ref	–	ref	–
Both social medical insurance and commercial medical insurance	−5.579 (−9.981–1.178)	0.013	−2.778 (−6.006–0.450)	0.092
**Household monthly per capita income**				
<2000	ref	–	–	–
2000–4999	−2.725 (−5.662–0.212)	0.069	–	–
5000–9999	1.999 (−0.072–4.070)	0.059	–	–
≥10000	0.827 (−1.908–3.562)	0.553	–	–
**The cost of purchasing enterostomy supplies mainly comes from**				
Medical insurance	70.144 (68.323–71.966)	<0.001	1.923 (−1.174–5.020)	0.223
Own expense	ref	–	ref	–
**Can I take care of my enterostomy now?**				
Completely unable to care	ref	–	ref	–
Partially care	6.969 (3.948–9.991)	<0.001	−0.676 (−3.264–1.912)	0.608
Completely care	5.409 (2.398–8.420)	<0.001	6.493 (4.036–8.950)	**<0.001**
**Duration of enterostomy**				
<6 months	Ref		ref	
6–12 months	1.334 (−1.476–4.143)	0.351	−1.430 (−3.680–0.820)	0.212
13–24 months	3.021 (0.380–5.663)	0.025	−2.081 (−4.490–0.329)	0.090
>24 months	7.826 (5.490–10.161)	<0.001	−0.455 (−2.954–2.043)	0.721
**enterostomy type**				
Temporary enterostomy	ref	–	ref	–
Permanent enterostomy	2.802 (1.196–4.409)	<0.001	1.511 (0.414–2.607)	**0.007**

All regression estimates are presented with 95% confidence intervals.

Bold values indicate statistical significance (*p* < 0.05).

Path analysis results demonstrated that knowledge directly influenced attitudes (β = 0.505, *P* = 0.023) and that attitudes directly influenced practices (β = 0.518, *P* = 0.006). While the direct effect of knowledge on practices was not significant (β = 0.151, *P* = 0.070), knowledge had a significant indirect effect on practices through attitudes, as confirmed by bootstrapping (indirect effect β = 0.262, 95% CI [0.089, 0.451], *P* = 0.011) (Supplementary Table 4).

## Discussion

### Knowledge

This subsection discusses the level of knowledge and its relationship with postoperative self-management practices among enterostomy patients. Patients with enterostomies exhibit adequate knowledge, positive attitudes, and proactive practices toward self-management of post-enterostomy. Because knowledge shapes attitudes and attitudes drive practices, interventions should prioritize knowledge building and attitude support to improve self-management.

The findings of this study reveal that patients with enterostomies generally possess sufficient knowledge, maintain positive attitudes, and engage in proactive practices regarding self-management post-enterostomy. Notably, patients with higher knowledge scores tend to exhibit more positive attitudes and proactive practices. These links are consistent with our multivariate results. This pattern is consistent with findings in other contexts, such as chronic disease management, where better knowledge positively correlates with improved self-care practice (23).

Several demographic and socioeconomic factors significantly influenced these dimensions. For instance, individuals residing in rural areas demonstrated higher levels of knowledge and more positive attitudes toward self-management compared to urban and suburban residents, echoing findings from previous research highlighting the potential impact of geographical location on health-related practice (24, 25). Similarly, individuals with lower educational attainment, specifically those with elementary education or below, exhibited higher scores across all dimensions. This finding contrasts with conventional wisdom, which often associates higher education levels with better health outcomes (26, 27). These individuals, owing to their potentially restricted health literacy and self-efficacy, may place greater reliance on healthcare professionals' advice and instructions. Consequently, this reliance could contribute to enhanced adherence to prescribed self-management practices. Furthermore, the duration of having an enterostomy was associated with better KAP, aligning with previous findings suggesting that longer experience with chronic disease may lead to improved self-management skills and coping strategies (28). Overall, knowledge appears to influence self-management by shaping patients' awareness, perceived control, and confidence in performing daily enterostomy care. These results underscore the interconnected nature of patients' KAP, prompting further analysis of their mutual influences.

### Attitudes

This subsection explores patients' attitudes toward enterostomy care and their influence on emotional adjustment, social reintegration, and adherence to self-management practice. The attitudes of respondents toward enterostomy care vary widely, with a significant portion expressing feelings of isolation, perceived disrespect, and concerns about social stigma. These findings align with prior research highlighting the psychological impact of living with an enterostomy. For example, a recent qualitative study exploring the experiences of patients with temporary stomas similarly identified significant burdens, anxieties about disease progression, and a profound need for emotional and social support (29). This underscores the critical importance of addressing patients' attitudes and emotional wellbeing as a core component of care. However, there are opportunities for improvement, as many respondents express a need for reassurance and a desire for equal treatment in relationships. To address these concerns, online support groups moderated by Mandarin-speaking healthcare professionals or enterostomy survivors can provide a safe space for patients to share experiences, seek advice, and receive emotional support. These groups can be hosted on platforms like WeChat or QQ, allowing users to engage anonymously if desired, thus mitigating concerns about privacy and stigma. Additionally, targeted educational campaigns aimed at reducing stigma and raising awareness about enterostomy-related issues within Chinese communities can be launched on social media platforms. Collaborating with influencers or celebrities who have openly discussed their experiences with enterostomy surgery can help amplify these messages and foster a more understanding and supportive environment. In summary, fostering positive attitudes is essential for improving patients' psychosocial adaptation and promoting sustained engagement in enterostomy self-care. Furthermore, translating improved attitudes into concrete self-care actions requires targeted behavioral interventions, which are discussed in the following section.

### Practices

This subsection examines patients' self-management practices and the factors influencing their ability to maintain consistent and effective enterostomy care. While respondents exhibit varying levels of self-care capability, there are notable gaps in practice, particularly regarding self-management tasks such as enterostomy bag replacement and identifying enterostomy abnormalities. Moreover, a significant proportion lacks consistency in seeking professional guidance when needed. This observation is consistent with a broader body of literature indicating that simply providing information is often insufficient. A recent literature review demonstrated that structured self-management education, utilizing various approaches such as telehealth and multimedia-based programs, is effective in improving patients' practical self-care abilities (30). Similarly, a randomized controlled trial found that a multimedia educational program significantly enhanced self-care ability and quality of life compared to conventional education (31). These findings strongly support our recommendation for implementing structured, skill-based educational programs. To bridge the gap in practical skills and promote proactive self-care practice among enterostomy patients in China, healthcare providers should offer comprehensive and culturally relevant educational programs (30, 32). This can include hands-on workshops led by experienced enterostomy nurses, covering topics such as proper bag replacement techniques, enterostomy care, and identifying potential complications. These workshops can be conducted in local hospitals or community centers and supplemented with online resources accessible via WeChat official accounts or dedicated websites. Additionally, implementing a telemedicine platform specifically for enterostomy patients can facilitate remote consultations with healthcare providers, enabling timely assistance and guidance when facing challenges with enterostomy care. Utilizing mobile applications that offer personalized care plans, reminders for medication and appointment schedules, and access to educational materials can further empower patients to take control of their health and adhere to recommended self-care practices (33, 34). Based on these gaps, we suggest structured education (pre-discharge and early post-discharge), scheduled telehealth follow-ups to troubleshoot skin or appliance issues, and routine screening for distress with referral to psychosocial support when needed.

### Integration and implications

The findings from the correlation and path analyses in our study highlight the interconnected nature of KAP among patients with enterostomies. The positive correlations and significant indirect effects point to the role of attitudes as a mediator between knowledge and practices. This indicates that increasing patients‘ knowledge may lead to improved attitudes, which in turn could enhance their management practices. This mechanistic pathway is not unique to ostomy care; similar patterns have been observed in other chronic conditions. For instance, a study on patients with type 2 diabetes found that a social media-based educational program effectively enhanced not only knowledge but also attitudes and self-care activities, with attitudes being a key driver of behavioral change (35). Our study validates this principle within the specific and challenging context of enterostomy self-management, where factors like body image and social stigma can make attitudes an even more potent determinant of practices. This concept aligns with well-established health behavior theories such as the Health Belief Model, which suggests that people's beliefs about health problems, benefits of action, and barriers to action can influence their health practice (36). In this case, improved knowledge might change patients' beliefs about the effectiveness and benefits of proper self-management, potentially leading to better practices.

Overall, respondents showed strong understanding of enterostomy care, including indications, basic diet advice, and bag-replacement steps. However, there are notable misconceptions regarding certain aspects, particularly concerning air travel restrictions for enterostomy patients. Additionally, awareness of common complications and initial post-surgery precautions falls slightly below optimal levels. To address this, healthcare providers should proactively provide accurate information during preoperative counseling and postoperative follow-up appointments (37, 38). Additionally, creating engaging and informative videos or infographics specifically addressing knowledge gap could be shared on popular Chinese social media platforms like WeChat and TikTok, reaching a wider audience and dispelling misconceptions effectively. Furthermore, to enhance awareness of common complications and initial post-surgery precautions, healthcare providers could develop easily accessible digital pamphlets or e-books in Chinese, detailing preventive measures and warning signs to watch for. Distributing these resources through hospital websites, patient portals, and social media channels can empower patients to take proactive measures in their care and seek timely medical assistance when needed (37, 39). In contexts where family involvement and collectivist values are prominent, relatives often share caregiving tasks and influence patients' beliefs and motivation. Incorporating family-inclusive education and aligning messages with community norms may therefore enhance attitude change and practice uptake.

Patterns observed in this study resemble KAP trends reported in other chronic conditions, such as diabetes and cardiovascular disease, where higher knowledge and positive attitudes are associated with improved self-care practice. However, enterostomy self-management presents unique challenges involving body image, appliance handling, and peristomal skin protection, which place greater emphasis on attitudes and practical skills. These differences highlight the novelty of this study and underscore the need for condition-specific, hands-on educational interventions in enterostomy care. Overall, these integrated insights emphasize the need for a comprehensive, patient-centered approach to enterostomy self-management that combines knowledge enhancement, attitudinal support, and skill-based education.

### Limitations

This study has several limitations. First, it must be noted that the questionnaire used in this study did not undergo formal construct validation. Consequently, the findings should be interpreted as a preliminary exploration of KAP levels rather than a definitive assessment of a psychometrically validated scale. Readers are cautioned against overinterpreting the underlying factor structure or latent relationships within the data. Future research is required to develop and formally validate a psychometrically robust instrument for this specific population. Second, the cross-sectional design precludes the establishment of causal relationships between variables; longitudinal studies would offer more robust insights into the dynamics of KAP over time. Third, the reliance on self-reported data may introduce response bias, potentially affecting the accuracy of the findings. Lastly, this study was conducted at a single institution with participants recruited from a specific geographical and cultural context in Beijing, which may limit the generalizability of the results to other regions in China or other countries. Given that cultural perceptions, social support systems, and healthcare accessibility can substantially influence patients' knowledge, attitudes, and self-management practices, caution should be exercised when generalizing these findings beyond similar sociocultural contexts. Future multi-center or international studies could provide a more comprehensive understanding of self-management patterns among enterostomy patients across diverse settings. Clinically, our findings support integrating brief attitude-focused counseling into routine education, screening for misconceptions before discharge, and providing follow-up touchpoints to reinforce self-management. Overall, enhancing practical self-care requires not only technical training but also continuous reinforcement through education and emotional support, effectively translating the KAP pathway into measurable improvements in daily care.

## Conclusion

In conclusion, patients with enterostomies exhibit adequate knowledge, positive attitudes, and proactive practices toward post-enterostomy self-management. Healthcare providers should prioritize interventions that enhance patients' attitudes, as these significantly influence self-care practices. Given the preliminary nature of this investigation and the lack of formal instrument validation, future multi-center studies utilizing standardized and psychometrically validated assessment tools are warranted to confirm these findings and establish causal relationships across diverse sociocultural contexts.

## Data Availability

The original contributions presented in the study are included in the article/Supplementary material, further inquiries can be directed to the corresponding authors.
