# Supplementary material for: Understanding patients' self-management after enterostomy: knowledge, attitudes, and practices in a cross-sectional study
Source: Front Public Health. 2026 Mar 18;14:1681498. doi: 10.3389/fpubh.2026.1681498 (PMC13038926; doi:10.3389/fpubh.2026.1681498)
Supplement: Supplementary file 1 [file Table_1.docx]

**Supplement Table 1. Distribution of knowledge dimension responses**

|  | **Correctness rate (%)** |
| --- | --- |
| 1. **Do you know the importance and necessity of ostomy?** | 491 (98.79) |
| 1. **Ostomy patients should avoid contact, heavy impact, or activities that increase abdominal pressure** | 472 (94.97) |
| 1. **Do you know the dietary guidance after ostomy surgery?** | 488 (98.19) |
| 1. **Do you know the normal color of stoma?** | 479 (96.38) |
| 1. **Do you know the steps to replace an ostomy bag?** | 489 (98.39) |
| 1. **Ostomy patients should take showers and try to control them within half an hour.** | 488 (98.19) |
| 1. **Ostomy patients cannot fly because they are under too much pressure and cannot clear their bowels in time.** | 241 (48.49) |
| 1. **Whether to provide guidance on the judgment of ostomy bag leakage and timing of replacement?** | 488 (98.19) |
| 1. **If the stoma chassis is cut too large, leakage may occur, causing fecal aqueous dermatitis.** | 495 (99.60) |
| 1. **If the stoma is cut too low, it can easily get stuck in the intestine, causing bleeding or ischemic necrosis.** | 496 (99.80) |
| 1. **If the stoma is slightly narrowed, the stoma can be expanded regularly under the guidance of medical staff.** | 489 (98.39) |
| 1. **Do you know about the common complications of stoma and surrounding areas?** | 400 (80.48) |
| 1. **Do you know the initial precautions after ostomy surgery?** | 483 (97.18) |

**Supplement Table 2. Distribution of attitude dimension responses**

| **Items, n (%)** | **Strongly agree** | **Agree** | **Neutral** | **Disagree** | **Strongly disagree** |
| --- | --- | --- | --- | --- | --- |
| **1. I feel isolated from healthy people.** | 10 (2.01) | 95 (19.11) | 20 (4.02) | 56 (11.27) | 316 (63.58) |
| **2. My illness makes me feel less respected than usual** | 29 (5.84) | 18 (3.62) | 29 (5.84) | 91 (18.31) | 330 (66.4) |
| **3. I feel that others may be worried about contracting my disease through contact with me, such as shaking hands with me or eating food prepared by me.** | 29 (5.84) | 17 (3.42) | 64 (12.88) | 51 (10.26) | 336 (67.61) |
| **4. I feel like others avoid me because of my illness.** | 10 (2.01) | 11 (2.21) | 62 (12.47) | 282 (56.74) | 132 (26.56) |
| **5. I worry about people telling others about my illness without my permission.** | 85 (17.1) | 63 (12.68) | 158 (31.79) | 64 (12.88) | 127 (25.55) |
| **6. I need reassurance that others care about me more than usual.** | 88 (17.71) | 10 (2.01) | 63 (12.68) | 212 (42.66) | 124 (24.95) |
| **7. Because of my illness, I feel like I am not equal in my relationships.** | 7 (1.41) | 12 (2.41) | 36 (7.24) | 122 (24.55) | 320 (64.39) |
| **8. Because of my illness, others seem embarrassed and nervous when they are around me.** | 3 (0.6) | 70 (14.08) | 42 (8.45) | 68 (13.68) | 314 (63.18) |
| **9. Because of my illness, I sometimes feel useless.** | 10 (2.01) | 73 (14.69) | 35 (7.04) | 258 (51.91) | 121 (24.35) |
| **10. Changes in my appearance have affected my social relationships.** | 8 (1.61) | 73 (14.69) | 34 (6.84) | 67 (13.48) | 315 (63.38) |

**Supplement Table 3. Distribution of practice dimension responses**

| **Items, n (%)** | **No** | **Rare** | **Sometimes** | **Often** | **Always** |
| --- | --- | --- | --- | --- | --- |
| **1. With the advice of the ostomy doctor, I can choose the appropriate ostomy bag and ostomy accessory products (such as anti-leakage cream, ostomy powder and skin protective film, etc.).** | 3 (0.6) | 1 (0.2) | 14 (2.82) | 197 (39.64) | 282 (56.74) |
| **2. I can replace the ostomy bag and accessories by myself.** | 50 (10.06) | 41 (8.25) | 6 (1.21) | 184 (37.02) | 216 (43.46) |
| **3. I am able to handle gas and feces in the ostomy bag by myself.** | 7 (1.41) | 1 (0.2) | 4 (0.8) | 192 (38.63) | 293 (58.95) |
| **4. I can clean the stoma and the surrounding skin myself.** | 39 (7.85) | 8 (1.61) | 3 (0.6) | 246 (49.5) | 201 (40.44) |
| **5. I know how to clean an ostomy bag.** | 35 (7.04) | 2 (0.4) | 11 (2.21) | 78 (15.69) | 371 (74.65) |
| **6. When I have a problem with my stoma care, I can find a solution.** | 3 (0.6) | 3 (0.6) | 93 (18.71) | 98 (19.72) | 300 (60.36) |
| **7. If given the opportunity, I would like to exchange stoma care information with patients with stoma.** | 47 (9.46) | 7 (1.41) | 14 (2.82) | 46 (9.26) | 383 (77.06) |
| **8. If there is anything I don’t understand about disease treatment and care, I will consult the doctor or nurse.** | 1 (0.2) | 2 (0.4) | 97 (19.52) | 87 (17.51) | 310 (62.37) |
| **9. I often read newspapers, go online or consult medical books to obtain knowledge about diseases.** | 29 (5.84) | 7 (1.41) | 56 (11.27) | 249 (50.1) | 156 (31.39) |
| **10. Regardless of whether there is any problem with my stoma, I will go to the stoma specialist clinic for regular check-ups.** | 24 (4.83) | 39 (7.85) | 63 (12.68) | 95 (19.11) | 276 (55.53) |
| **11. I can judge by myself whether there are abnormalities or complications with the stoma.** | 6 (1.21) | 40 (8.05) | 100 (20.12) | 84 (16.9) | 267 (53.72) |
| **12. I usually pay attention to eating less foods that can easily cause diarrhea, constipation and flatulence.** | 24 (4.83) | 7 (1.41) | 63 (12.68) | 93 (18.71) | 310 (62.37) |
| **13. I will choose appropriate exercise methods according to my physical condition.** | 25 (5.03) | / | 14 (2.82) | 128 (25.75) | 330 (66.4) |
| **14. I usually avoid lifting heavy objects or hitting the stoma directly.** | 1 (0.2) | 2 (0.4) | 35 (7.04) | 131 (26.36) | 328 (66) |
| **15. I can control the negative emotions (such as anger, fear, etc.) caused by having an ostomy.** | 3 (0.6) | 1 (0.2) | 13 (2.62) | 146 (29.38) | 334 (67.2) |
| **16. I will try my best to change the idea that I am a burden to my family.** | 3 (0.6) | 11 (2.21) | 12 (2.41) | 191 (38.43) | 280 (56.34) |

**Supplementary Table 4 Path analysis results**

|  | | | **Standardized total effects** | | **Standardized direct effects** | | **Standardized indirect effects** | |
| --- | --- | --- | --- | --- | --- | --- | --- | --- |
|  |  |  | **Estimates (95% CI)** | **P** | **Estimates (95% CI)** | **P** | **Estimates (95% CI)** | **P** |
| A-total | <--- | K-total | 0.505 (0.412-0.585) | 0.023 | 0.505 (0.412-0.585) | 0.023 |  |  |
| P-total | <--- | A-total | 0.518 (0.420-0.647) | 0.006 | 0.518 (0.420-0.647) | 0.006 |  |  |
| P-total | <--- | K-total | 0.412 (0.287-0.493) | 0.023 | 0.151 (-0.063-0.290) | 0.070 | 0.262 (0.190-0.350) | 0.011 |
